# Variants of the *ABCA3* gene might contribute to susceptibility to interstitial lung diseases in the Chinese population

**DOI:** 10.1038/s41598-017-04486-y

**Published:** 2017-06-22

**Authors:** Wei Zhou, Yi Zhuang, Jiapeng Sun, Xiaofen Wang, Qingya Zhao, Lizhi Xu, Yaping Wang

**Affiliations:** 1 Department of Medical Genetics, Nanjing University School of Medicine, Nanjing, China; 2 Department of Respirology, Medical School Affiliated Drum Tower Hospital, Nanjing University, Nanjing, China; 3 Jiangsu Key Laboratory of Molecular Medicine, Nanjing University School of Medicine, Nanjing, Jiangsu China

## Abstract

ATP-binding cassette A3 (ABCA3) is a phospholipid carrier that is mainly expressed in the alveolar epithelium. Biallelic mutations of *ABCA3* has been associated with fatal respiratory distress syndrome and interstitial lung disease (ILD) in children. However, whether variations in ABCA3 have a role in the development of adult ILD, including idiopathic pulmonary fibrosis (IPF), remains to be addressed. In this study, we screened for germline variants of *ABCA3* by exons-sequencing in 30 patients with sporadic IPF and in 30 matched healthy controls. Eleven missense variants, predominantly in heterozygous, were found in 13 of these patients, but only two missenses in 2 healthy controls. We then selected four of the detected missense variants (p.L39V, p.S828F, p.V968M and p.G1205R) to performed cohort analysis in 1,024 ILD patients, containing 250 IPF and 774 connective tissue disease-ILD (CTD-ILD) patients, and 1,054 healthy individuals. Our results showed that the allele frequency of p.G1205R, but not p.L39V, was significantly higher in ILD patients than in healthy controls. However, no additional subject carrying the variant p.S828F or p.V968M was detected in the cohort analysis. These results indicate that the heterozygous *ABCA3* gene variants may contribute to susceptibility to diseases in the Chinese population.

## Introduction

The ATP-binding cassette (ABC) transporter is an essential membrane protein involved in the transport of compounds across the biological membranes. Dysfunction of the genes encoding ABC transporters has been associated with several human genetic diseases. Tangier disease, characterized by the abnormal accumulation of cholesterol in body tissues and a deficiency of high-density lipoproteins, is caused by mutations of the *ABCA1* gene^1^. Mutations in *ABCA4* are responsible for Stargardt’s disease, a type of macular degeneration associated with a severe loss of central vision^2^. *ABCA12* mutants result in an autosomal recessive congenital ichthyosis via defective lipid transport in the keratinocytes^3^. Biallelic mutations of *ABCA3* (OMIM: 601615) have been associated with surfactant deficiencies and respiratory diseases in infants and with interstitial lung diseases in children and adults^4, 5^. The ABCA3 protein is expressed in a series of tissues and is highly expressed in alveolar epithelial type II cells. It is localized to the limiting membrane of the lamellar body, where it plays an important role in pulmonary surfactant synthesis and transport^6^. The *ABCA3* gene is located on chromosome 16q13.3 and encodes a polypeptide of 1,704 amino acids with 2 homologous repeats, each harboring a nucleotide-binding domain and a membrane-spanning domain. To date, more than 100 mutations have been identified in the *ABCA3* gene. Some of these mutations have been reported to induce impaired processing and intracellular mislocalization of the protein^7^. They change the structure of ABCA3 protein, affect the maturation of the lamellar body and destroy the homeostasis of alveolar surfactants^8, 9^.

Interstitial lung disease (ILD) is a heterogeneous collection of many pulmonary disorders that affect the tissue and spaces surrounding the alveoli. ILD causes an irreversible architectural distortion and then impairs gas exchange^10^. A group of ILDs presents with underlying connective tissue diseases (CTD), including systemic sclerosis (SSc), rheumatoid arthritis (RA) and inflammatory myositis, and these types of ILD are referred to CTD-ILD^11^. Idiopathic pulmonary fibrosis (IPF) represents a specific form of ILD characterized by pulmonary fibrosis or progressive alveolar interstitial lesions with an unknown cause. IPF occurs primarily in elderly people and is associated with a poor prognosis. The median survival for patients affected by IPF varies from 2 to 5 years, and the patients exhibit variable disease courses and prognoses^12^.

Although the direct mechanism underlying IPF is not completely understood, a genetic predisposition has been considered one of the important causes of this disease. Mutations in the surfactant protein associated genes *SFTPC* and *SFTPA2* have been identified in some of the familial cases of pulmonary fibrosis^13^. Campo I^5^ and Coghlan M *et al*.^14^ reported that homozygous or compound heterozygous mutations in *ABCA3* might also be related to adult-onset fibrotic lung disease. In this study, we first screened for variations of *ABCA3* in a set of patients with sporadic IPF, and then a cohort analysis was performed to assess ILD susceptibility among the subjects carrying the detected variants. We found that the heterozygous *ABCA3* gene variants may contribute to susceptibility to interstitial lung diseases in the Chinese population.

## Results

Table 1 shows the general characteristics of the subjects in our study.

**Table 1:** Characteristics of the investigated subjects.

	Characteristics^#^	Number	Male	Female	Mean Age
Section 1*	Healthy controls	30	15	15	66.7 ± 4.3
	IPF	30	15	15	66.7 ± 4.3
Section 2*	Healthy controls	1054	649	405	55.6 ± 15.0
	ILD	1024	543	481	60.1 ± 13.5
	CTD-ILD	774	316	458	58.8 ± 12.9
	IPF	250	227	23	66.9 ± 10.4
	Pneumonias	109	69	40	62.9 ± 18.1

*The subjects of section 1 were recruited for investigation of the *ABCA3* gene by exons-sequencing; The subjects of section 2 were recruited for cohort analysis; ^#^ILD: Interstitial lung diseases; IPF: Idiopathic pulmonary fibrosis; CTD: connective tissue diseases.

### Variants detected in the *ABCA3* gene by exons-sequencing

We screened for variants of the *ABCA3* gene exon by exon by DNA sequencing among thirty of the sporadic IPF patients and thirty of the healthy controls, respectively. Eleven distinct missense variants were detected in thirteen IPF patients and two missenses in two healthy controls. The detection rate of missense variants in the *ABCA3* gene in the IPF patients was significantly higher than that in the healthy controls (P = 0.002, OR = 10.71, 95%CI: 2.15–53.35). Two distinct synonymous variants were observed in four IPF patients and in three healthy controls, respectively. No significant different was found between the two groups on the detection rate of synonymous variants (Table 2). Most of the variants, whether missense or synonymous, presented as heterozygous. However, three IPF patients were found to have two different variants: P-22 carried two missense variants, and P-1 and P-12 both had one missense and one synonymous variant, respectively. Table 2 shows the information on the fifteen variants detected in the exons of the *ABCA3* gene in IPF patients and in healthy controls. No hotspot mutation reported formerly in neonatal respiratory distress syndrome (NRDS) was found in these samples. Twelve of the detected variants had been described in dbSNP (http://www.ncbi.nlm.nih.gov/snp/), and the other three were novel (Table 3). Additionally, seventeen distinct single base pair substitutions were also found in the introns sequences adjacent to exons of the *ABCA3* gene. We show these in the supplement (see Supplementary Table S1).

**Table 2:** The variants detected in *ABCA3* gene by sequencing in 30 sporadic IPF patients and 30 healthy controls.

Types	*No. of subject	Gender	Age	Smoking	Exon	Nucleotide alteration	Amino acid change	^&^Detection rate	^§^P value
Missense variants	P-1	M	69	Yes	15	c.1809G > C	p.Q603H	13/30	0.002
	P-2	F	65	No	33	c.5020 G > A	p.G1674S		
	P-3	F	70	No	5	c.277 G > A	p.V93I		
	P-7	M	63	Yes	24	c.3613 G > A	p.G1205R		
	P-9	M	70	Yes	19	c.2483 C > T	p.S828F		
	P-10	F	62	No	5	c.115 C > G	p.L39V		
	P-12	M	69	No	15	c.1809G > C	p.Q603H		
	P-14	M	69	Yes	22	c.3060 T > A	p.N1020K		
	P-16	M	60	No	5	c.115 C > G	p.L39V		
	P-19	F	68	No	18	c.2377 G > A	p.E793K		
	P-21	F	74	No	24	c.3613 G > A	p.G1205R		
	P-22	M	64	No	8	c.869 T > A, c.2902 G > A	p.L290Q		
					21		p.V968M		
	P-26	F	60	No	33	c.4993 A > G	p.I1665V		
	H-12	F	65	No	16	c.1913G > A	p.R638H	2/30	
	H-14	F	68	No	16	c.2032 G > A	p.A678T		
Synonymous variants	P-1	M	69	Yes	15	c.1755C > G	p.P585P	4/30	1.000
	P-4	M	63	Yes	5	c.213 C > T	p.F71F		
	P-12	M	69	No	5	c.213 C > T	p.F71F		
	P-18	F	60	No	15	c.1755C > G	p.P585P		
	H-7	M	64	Yes	15	c.1755C > G	p.P585P	3/30	
	H-24	M	69	Yes	15	c.1755C > G	p.P585P		
	H-29	F	73	No	5	c.213 C > T	p.F71F		

*P: Patient number; H: Healthy control number; ^&^Number of carriers detected in 30 IPF patients or 30 healthy controls; ^§^A comparison between the rates of variant carriers in 30 IPF patients and 30 healthy controls (Fisher’s exact test).

**Table 3:** The predicted effect of the variants on protein function with bioinformatics assay.

Variations	dbSNP ID	MAF		SIFT	PolyPhen2	Pon-P2
		1000 G*	ExAC^&^			
p.L39V	rs200090198	0.0002	0.003	Deleterious	Possibly damaging	Unknown
p.F71F	rs117515055	0.0056	0.0317	NA^†^	NA	NA
p.V93I	rs199840288	0.0004	0.003	Tolerated	Benign	Neutral
p.L290Q	NA			Deleterious	Probably damaging	Unknown
p.P585P	rs323043	0.0817	0.2018	NA	NA	NA
p.Q603H	rs753223759			Tolerated	Benign	Neutral
p.R638H^#^	rs145269995	0.0008	0.002	Tolerated	Benign	Neutral
p.A678T^#^	rs769584477			Deleterious	Possibly damaging	Unknown
p.E793K	rs760598605			Deleterious	Possibly damaging	Unknown
p.S828F	NA			Deleterious	Probably damaging	Unknown
p.V968M	rs150340257	0.0004	0	Deleterious	Probably damaging	Unknown
p.N1020K	NA			Deleterious	Benign	Unknown
p.G1205R	rs549977217	0.0002	0.007	Tolerated	Benign	Unknown
p.I1665V	rs781259566			Tolerated	Benign	Neutral
p.G1674S	rs202078936	0.0006	0.003	Tolerated	Benign	Neutral

*Minor allele frequency (MAF) from 1000 Genomes Project (1000 G); ^&^MAF from Exome Aggregation Consortium (ExAC) in East Asians; ^†^NA: not available; ^#^The two variants were detected in the healthy controls.

### Characterizations of *ABCA3* variants

**Figure 1:**
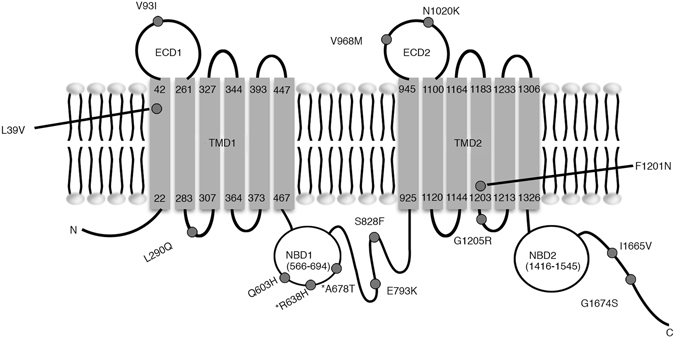
Structural diagram of the ABCA3 protein and the distribution of the missense variants detected in ABCA3 in this study. *The two variants were detected in the healthy controls.

The ABCA3 protein consists of two tandem functional units, i.e., N-half and C-half. Both of these consist of a six-unit transmembrane complex (TMC) and an nt-binding domains (NBD)^15^. Many functional mutations reported previously tended to cluster in the extracellular region of the cell membrane (ECD) and the NBD. Three of missense variants detected in this study (p.V93I, p.V968M and p.N1020K) were in the ECD, and three (p.Q603H, p.R638H and p.A678T) was in NBD1. The distribution of the thirteen missense variants in ABCA3 is shown in Fig. 1.

We then applied three of the variants prediction algorithms, i.e., SIFT, PolyPhen and Pon-P2, which use different parameters, to assess the functional damage arising from the detected thirteen missense variants. Seven of the missense variants (p.L39V, p.L290Q, p.A678T, p.E793K, p.S828F, p.V968M and p.N1020K) could be deleterious according to the three prediction algorithms, including one (p.A678T) detected in the healthy controls. One (p.G1205R) was predicted to potentially affect the function of the ABCA3 protein according to the algorithm Pon-P2 (Table 3).

A recent study showed that more than half of all point mutations responsible for genetic diseases could cause aberrant splicing^16^. Synonymous variants could interrupt exon splicing regulation and had dramatic effects on the structure of the gene product. Using SR protein score matrices and threshold values, we evaluated the potential exonic splicing enhancers (ESE) motifs of the sequences containing the detected variants in the *ABCA3* gene (listed in Table 4). Eleven of the variants (73.33%) were predicted to result in abrogation and/or addition of the ESE motifs. The variant c.1809G > C (p.Q603H) was predicted to abrogate the motif response to SF2/ASF but to add to the motifs responses to the other three SR proteins (SC35, SRp40 and SRp55).

**Table 4:** Alterations of the putative ESE motifs with these variants detected in sporadic IPF patients and healthy controls.

Exon	Nucleotide change	ESE-finder*			
		SF2/ASF	SC35	SRp40	SRp55
5	c.115 C > G		−2/2		
5	c.213 C > T				
5	c.277 G > A	+ 1/0			
8	c.869 T > A	+2/0	−1/1		
15	c.1755C > G				
15	c.1809G > C	−1/1	+1/0	+1/0	+1/0
16	c.1913G > A^#^				
16	c.2032 G > A^#^				
18	c.2377 G > A	−2/2			
19	c.2483 C > T				−1/1
21	c.2902 G > A	−1/1			
22	c.3060 A > T			−1/1	
24	c.3613 G > A			+1/0	
33	c.4993 A > G			−1/1	
33	c.5020 G > A		+1/0		

*Number of ESE motifs added or abrogated from the variant/number of ESE motifs in the normal allele; ^#^The two variants were only detected in the healthy controls.

### Analysis of association of the variants with the risk of ILD

**Figure 2:**
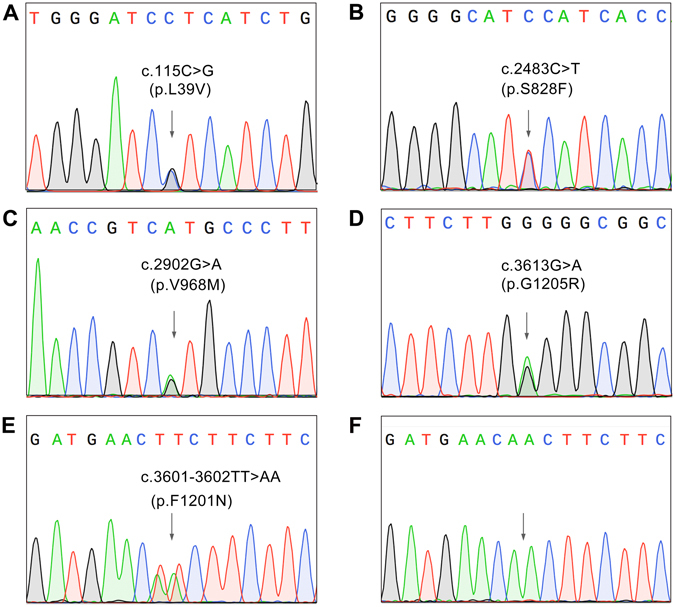
Confirmation sequencing of the variants detected in the *ABCA3* gene, by using a TaqMan probe. (**A,B,C,D)** the variants c.115 C > G (p.L39V), c.2483 C > T (p.S828F), c.2902 G > A (p.V968M) and c.3613 G > A (p.G1205R) are shown. (**E)** the two base pairs substitution, c.3601-3602TT > AA, in the exon 24 in *ABCA3*. (**F**) Cloning sequencing confirmed that the substitution of two base pairs occurred in the same copy of the *ABCA3* gene, that results in p.F1201N.

To evaluate the phenotypic effects of the variants, we chose four missense variants, i.e., p.L39V, p.S828F, p.V968M and p.G1205R for cohort analysis in our recruited ILD patients and healthy controls (Fig. 2A,B,C and D). The variants p.L39V and p.G1205R were detected in 2 of 30 of the IPF patients by exons-sequencing of the *ABCA3* gene, respectively. The functional predictions showed that the variants p.L39V would be deleterious by three of the variants prediction algorithms, SIFT, PolyPhen and Pon-P2, and the variant p.G1205R might affect the function of ABCA3 protein with the Pon-P2. The variants p.S828F and p.V968M were detected only in 1 of 30 of the IPF patients respectively, and the p.S828F was a novel variant found in this study. Both of the variants was likely to be damaging, as shown by the functional predictions. We used the TaqMan probes to determine the genotypes of *ABCA3* for the variants in 1,024 patients with ILD (the 30 sequenced subjects mentioned were not included), 109 patients with community acquired pneumonia and 1,054 healthy controls. Our results showed that the allele frequency of p.G1205R was significantly higher in ILD patients than in healthy controls (Table 5). This indicated that the minor allele for this variant of the *ABCA3* gene was associated with risk of ILD. However, a higher allele frequency of p.G1205R was only detected in CTD-ILD patients when we made a comparison between the subgroups of ILD patients and healthy controls (Table 6). On the other hand, no significant difference was found on the allele frequency of the variant p.L39V between the ILD patients and the healthy individuals. The variant p.G1205R, but not p.L39V, was also found to be prevalent in pneumonia patients compared to healthy individuals (Table 5). We tested the variants p.S828F and p.V968M in the ILD patients, pneumonia patients and healthy people. No additional subject carrying these variants was detected among the three groups. Interestingly, a novel heterozygous c.3601-3602TT > AA variant was unexpectedly found in one of the IPF patients for whom we did sequencing to validate the genotype suspected for p.G1205R according to the TaqMan probe assay. Cloning sequencing showed that the substitution of two base pairs occurred at the same copy of the *ABCA3* gene, that resulted in p.F1201N. This variant was in the second transmembrane region of the ABCA3 protein. Bioinformatics analysis indicated that p.F1201N in ABCA3 would cause a functional impairment of this protein (Figs. 1 and 2E,F).

**Table 5:** Comparisons of allele frequencies on the variants p.L39V and p.G1205R in ABCA3 between ILD patients and healthy controls^#^.

Variations	Groups	No. of allele		P-value	OR^†^(95% CI^§^)
		Wild-type allele	Variant allele		
p.L39V	Control	2105	3		
	ILD	2044	4	0.446	2.06(0.38–11.26)
	Pneumonias	218	0	1.000	0.99(0.99–1.00)
p.G1205R	Control	2106	2		
	ILD	2038	10	0.021	5.17(1.13–23.61)
	Pneumonias	214	4	0.001	19.68(3.58–108.08)

^†^odds ratio; ^§^95% confidence interval; ^#^Bonferroni correction was used to adjust the significance level to 0.025.

**Table 6:** Comparisons on the variants p.L39V and p.G1205R in ABCA3 between the different subgroups of ILD patients and healthy controls^#^.

Variations	Groups	No. of allele		P-value	OR^†^(95% CI^§^)
		Wild-type allele	Variant allele		
p.L39V	Control	2105	3		
	CTD-ILD	1546	2	1.000	0.91(0.15–5.45)
	IPF	498	2	0.246	2.83(0.47–17.00)
p.G1205R	Control	2106	2		
	CTD-ILD	1540	8	0.022	5.49(1.16–25.94)
	IPF	498	2	0.168	4.24(0.60–30.26)

^†^odds ratio; ^§^95% confidence interval; ^#^Bonferroni correction was used to adjust the significance level to 0.025.

### Gender-stratified analysis of variants in ILD patients

To investigate whether gender affects the risk of ILD as a function of the variants in the *ABCA3* gene, an analysis stratified by gender was performed for L39V and G1205R. There was no difference in the rate at which the two variants were detected in male and female subjects either in the healthy control group or in the ILD patient group (data not shown).

## Discussion

ILD encompasses a heterogeneous group of parenchymal lung disorders characterized by diverse pathogenesis and complex histopathology. The onset of ILD has been correlated with exposure to many types of triggers and with well-characterized diseases, but the trigger mechanisms of ILD remain elusive. It is now clear that the development of ILD has a strong genetic basis. Deficiencies in the genes encoding the lung surfactant proteins have been associated with ILD. Multiple disease-causing mutations of surfactant protein C (*SFTPC)* have been reported in an autosomal dominant manner in the familial ILD with variable penetrance^17, 18^. The most common *SFTPC* mutation, a substitution of isoleucine with threonine at codon 73 (p.I73T), accounting for approximately 30% of all cases, was reported in both sporadic and inherited cases^19, 20^. The gene encoding the surfactant protein *SFPTA2* has also been considered a candidate because of its key role in alveolar stability, and mutations of this gene have been identified in familial pulmonary fibrosis (FPF) kindred^21^. Mutations in four telomere-related genes, i.e., *TERT*, *TERC*, *RTEL1* and *PARN*, have also been linked to a variety of ILD types, including IPF, CTD-ILD and other types of interstitial pneumonia^22^. Approximately 15% of familial IPF and 5% of sporadic cases have been found to carry heterozygous mutations in the *TERT* or *TERC* genes^23, 24^. Additionally, genome-wide linkage analysis has revealed an association between both familial and sporadic IPF and a single nucleotide polymorphism (SNP) rs35705950 in the promoter region of the *MUC5B* gene^25, 26^. The minor allele of *MUC5B* has been detected at a frequency of 34% among patients with familial interstitial pneumonia and 38% among patients with sporadic IPF, but 9% among healthy controls. These results strongly indicate that the minor allele has a substantial effect on the pathogenesis of pulmonary fibrosis in the Western population^12^. The association between polymorphism rs35705950 and ILD has also been confirmed in Chinese populations^27^, but the frequency of this allele is much lower than in Caucasians. The genetic basis of ILD in the Chinese population remains to be addressed.

Homozygous or compound heterozygous mutations of the *ABCA3* gene are currently considered the etiological basis of severe neonatal lung diseases and symptoms of surfactant deficiency. Recently, the *ABCA3* mutations were also identified in young adults with ILD^28^ and even in adults with IPF and emphysema^29^. However, there are no data showing whether the deficiency of ABCA3 is prevalent in ILD development in the Chinese population. We screened for all of the coding sequences in the *ABCA3* gene in 30 sporadic IPF patients and the matched healthy controls. Moreover, we found eleven distinct missense variants in thirteen patients and two in healthy controls. Of the thirteen-detected missense variants, six had been reported with an allele frequency that is less than 1% for each of them, four have been reported but with no data on their allele frequency, and three are novel with no available information on their allele frequencies. The minor allele frequency we consulted here were validated by the 1000 Genomes Project (1000 G) and Exome Aggregation Consortium (ExAC; http://exac.broadinstitute.org) (shown in Table 3). These data indicate that all of the missense variants detected in this study could be rare variants. The ExAC data suggest that the carrier rate of rare variations in *ABCA3* gene is closer to 4.5% among East Asians. However, the minor allele frequencies of the variants in ExAC contained the data from exome sequencing of the patients with adult-onset diseases. Chen, *et al*. recently reported that the carrier rate of functional rare variations in the *ABCA3* gene is approximately 1.3% in the Chinese population, and this rate could be 3%-5% in European or African populations^30^. The subjects with rare missense variants of the *ABCA3* accounted for 43% (13/30) of the sporadic IPF patients in our study. It is significantly higher than in the matched controls (2/30). Bioinformatics analysis with three variant prediction algorithms suggested that six of the missense variants (p.L39V,,p.L290Q, p.E793K, p.S828F, p.V968M, p.N1020K) could affect the function of the ABCA3 protein in 7/30 of the IPF patients, but only one (p.A678T) in 1/30 of the healthy controls. The score matrices for the SR protein binding motif showed that eleven of the single base pair substitutions among the fifteen missense and synonymous variants in this work would add to or abolish at least one of the potential ESE motifs, including the variant p.G1205R, which we employed in cohort analysis for evaluation of ILD risk.

As a member of the ABC transporter family, ABCA3 hydrolyzes ATP to transport choline-phospholipids and cholesterol into lamellar bodies in alveolar epithelial type II cells^6^. Functional *ABCA3* variants can lead to abnormal intracellular localization of the ABCA3 protein and to alteration of ABCA3’s functions with reduced ATPase activity or impaired phospholipid transport^7^. Increasing evidence has indicated that the variations clustering in the ECD and NBD domains of ABCA3 could alter protein folding/localization and cause functional impairments. Matsumura Y and Cheong N, *et al*. reported that the proteins with variations in the ECD exhibited the most severe impairment of intracellular traffic and were predominantly localized at the ER^7, 31^. Weichert N showed that the variants in the transmembrane domain resulted in retention of the ABCA3 protein in ER and would elevate ER stress^32^. The protein mutants occurring in the intracellular loop and NBD domain were described as having normal trafficking and protein processing but decreased ATP hydrolysis activity^7, 33, 34^. Of the missense variants detected in this study, including p.F1201N (which was unexpectedly found with the TaqMan probe), the three variants that occurred at ECD (one at ECD1 and two at ECD2) were detected in the IPF patients. In the three variants that occurred at NBD1, one was observed in the IPF patients and two in the healthy controls. That could indicate a different functional impairment caused by these variants at the domains of ABCA3 protein. (Fig. 1). Whether these variants affect the incidence of ILD in the Chinese population needs to be assessed further. We selected four variants (p.L39V, p.S828F,p.V968M and p.G1205R) to carry out a cohort analysis among ILD patients, pneumonia patients and healthy individuals. The four variants occurred at different functional domains of the ABCA3 protein. Both p.L39V and p.G1205R were detected in two cases, p.S828F and p.V968M were detected in one patient respectively, when we screened for germline variants by exons-sequencing in 30 sporadic IPF cases. Functional evaluations with bioinformatics suggested that the variants p.L39V, p.S828F and p.V968M would be deleterious by the three variants prediction algorithms, and the variant p.G1205R was a suspicious functional variation by one of the algorithms. Moreover, the single base pair substitution resulting in p.G1205R could add one ESE motif in exon 24 of the *ABCA3* gene. The variant p.S828F was a novel detected in this study.

Our results showed that carriers with p.G1205R in *ABCA3* were susceptible to ILD, especially to CTD-ILD. The variant p.G1205R was also associated with the risk of pneumonias. However, no significant different on the allele frequency of p.L39V was found between the ILD patients and the healthy controls. Additionally, we did not identify any additional instances of p.S828F and p.V968M in the subsequent cohort analysis, either in ILD patients or in healthy controls. This indicated that these two variants in ABCA3 were accidental variants in IPF patients.

The majority of IPF patients are elderly males with a history of smoking; however, IPF is sometimes observed in people who have never smoked^35, 36^. Of note, IPF patients who have never smoked can develop a more acute exacerbation (AE) and have a poorer prognosis than smokers^37^. An epidemiological investigation showed that more than half of all Chinese men are smokers, but less than 3% of Chinese women smoke^38^. The information on the ILD patients in this study indicated that approximately 60% of the male ILD patients had been smokers, but none of the women patients had ever smoked (some of IPF data are shown in Table 2). However, the frequencies of the two variants in the *ABCA3* gene (p.L39V and p.G1205R) did not differ between males and the females in our study, either in the healthy individuals group or in the ILD patients groups.

An autosomal recessive pattern of ABCA3 deficiency has been described in neonatal respiratory distress syndrome and was recently extended to fibrotic lung disease in middle-aged and elderly population. However, the rare missense variants of the *ABCA3* gene detected in this study predominantly presented as heterozygous in ILD, in IPF or CTD-ILD patients, although one of the IPF patients carried two missense variants of ABCA3 (Table 2). This suggests that the heterozygous *ABCA3* variants could contribute to susceptibility to diseases in the Chinese population. We consider that the genetic susceptibility to ILD could be due to a haploid deficiency of the ABCA3 gene. Whether the “two-hit” model, on that the alveolar epithelium cells with the impaired *ABCA3* gene are constantly accumulated with ageing, plays a role remains to be addressed.

The results of this study were obtained from a preliminary investigation of variants in the *ABCA3* gene. The rare missense variants showed by exons-sequencing among IPF patients and healthy controls were taken from a limited sample size in this study. The prevalence of *ABCA3* variants in ILD patients should therefore be validated with multicenter investigations and more accurate statistical analysis and with further functional characterization of these variants.

## Materials and Methods

### Ethics Statement

The study protocol was reviewed and approved by the ethics committee and review boards of Nanjing University School of Medicine. The methods were carried out in accordance with the approved guidelines and regulations. Written informed consent was obtained from all of the recruited patients and control subjects before any study procedure were performed.

### Subjects

All the patients and healthy controls were recruited from the Affiliated Drum Tower Hospital, Nanjing University School of Medicine, China. The ILD patients were consecutively recruited from 2007 to 2015 with the following clinical data recorded: age, gender, family history, past medical history, smoking history, occupational exposure history, physical examination findings, and laboratory results. All of the recruited ILD patients were diagnosed on the basis of clinical features and a high-resolution computed tomography (HRCT) evaluation by respiratory specialists. A total of 1,054 ILD patients were categorized into groups according to their clinical features and the 2011 ATS/ERS consensus: 280 with IPF (exclusion of the known causes of interstitial lung disease) and 774 with autoimmune connective tissue disease-associated ILD (CTD-ILD). The ILD patients with autoimmune connective tissue diseases, including Sjögren’s syndrome (SS), rheumatoid arthritis (RA) and systemic lupus erythematosus (SLE), were also diagnosed by rheumatologists in the same hospital. We randomly recruited 1,084 healthy individuals as controls from people who attended the same hospital for a routine health examination. Subjects who suffered from acute inflammation, tuberculosis, autoimmune disease or cancers were excluded. These ILD patients and healthy individuals were recruited in two sections for the germline variation screening of *ABCA3* gene and for the cohort analysis on part of the detected variations, respectively. Additionally, we recruited 109 pneumonia patients who had been excluded as having ILD as a control group for pulmonary disease cases (Table 1). These patients had community acquired pneumonia that was diagnosed by respiratory specialists based on their symptoms, sputum bacterial culture, laboratory tests and chest radiography/computed tomography.

### DNA extraction

Peripheral venous blood samples from all the subjects above were collected in EDTA-containing anticoagulant. Genomic DNA was extracted from the blood using a TIANamp Genomic DNA Kit (TIANGEN), according to the manufacturer’s protocol, and was quantified by spectrophotometry.

### Variant screening of *ABCA3* gene in sporadic IPF patients and healthy controls

We randomly recruited 30 sporadic IPF patients (15 males and 15 females) and 30 age and sex matched healthy controls for screening for germline variations of the *ABCA3* gene in their genomic DNA. We designed 25 primer pairs for polymerase chain reaction (PCR) of the 30 coding regions (exons 4~33) and the intron-exon boundaries of the *ABCA3* gene (see Supplementary Table S2). The 25 μL PCR volume contained 100 ng of genomic DNA, 12.5 μl of Master Mix (Vazyme, China), and 25 pmol of each primer, and the remaining volume was filled with autoclaved reverse-osmosis-purified water. The PCR reactions were performed for 35 cycles at 95 °C for 1 minute, 60 °C (dependent on the primer sequences) for 30 s, 72 °C for 30 s, and finally 72 °C for 5 minutes to complete the extension. The purified PCR products were directly sequenced using an ABI BigDye Terminator v3.1 Cycle Sequencing Kit. The analyses were completed on a 3130 Genetic Analyzer (Applied Biosystems).

### Bioinformatics analysis of the detected variants

The web-based tools SIFT, PolyPhen, PON-P2 and ESEfinder were used to make a preliminary functional evaluation of the variants detected in the 30 sporadic IPF patients and in the 30 healthy controls. The Sorting Intolerant from Tolerant: (SIFT; http://sift.jcvi.org/) algorithm was used to predict the effect of an amino acid substitution on protein function. The scores ranged from 0 to 1, where 0 denotes damaging and 1 denotes neutral^39^. For the PolyPhen-based prediction (http://genetics.bwh.harvard.edu/pph2/), the results shown as benign would be considered nonfunctional variants, while those found to be possibly or probably damaging were considered functional^40^. PON-P2 (http://structure.bmc.lu.se/PON-P2/) is a new computational profiling tool for classifying the amino acid substitutions resulting from DNA variations: it classifies these into pathogenic, unknown and neutral based on the forecast probability score^41^. ESEfinder was used to predict whether the exonic variations could disrupt or produce putative exonic splicing enhancers (ESE), which are response elements for the human SR proteins (SF2/ASF, SC35, SRp40 and SRp55^42^, which are involved in pre-mRNA splicing). The program can score the input sequences, and the scores above a default threshold value were predicted to act as ESEs (ESEfinder3.0, http://rulai.cshl.edu/cgi-bin/tools/ESE3/esefinder.cgi).

### Cohort analysis on the detected variants

Some of the *ABCA3* variants detected in the 30 sporadic IPF patients had cohort analysis performed on them, with a comparison between ILD patients and healthy individuals in a larger sample set. Real-time PCR with the TaqMan probe (Applied Biosystems, Foster City, CA) was used to genotype the selected variants in the recruited ILD (IPF and CTD-ILD) patients, pneumonias patients and healthy controls. We used a 10 μl reaction system containing 2.5 μl of Taqman universal genotyping master mix, 0.125 μl of Taqman 20 × SNP assay, 1.5 μl of DNA (5 ng/μl), with the remaining volume consisting of autoclaved reverse-osmosis-purified water. The reactions were performed using a Viia7 Fast Real-time PCR system (Applied Biosystems). The PCR cycle included a 95 °C 10-minute hot start, followed by 40 cycles of two-step PCR (15 seconds at 95 °C for denaturing and 1 minute at 60 °C for annealing and extension). Digital PCR analysis software (TaqMan GenoTyper v1.3) was used to process the data. To validate the genotyping results of the TaqMan assay, we performed sequencing on all of the samples that had been assessed to carry the checked variations in *ABCA3*, and more than ten samples were shown to be non-carriers.

### Statistical analysis

The statistical program SPSS version 19.0 was used to carry out the statistical analysis of the associations between the genotypes and disease status. We performed Fisher’s exact test to compare the frequencies of the variants between the patient and control groups (*P* < 0.05 was considered statistically significant). For risk assessment, we used an unconditional logistic regression to calculate the odds ratios (OR) and 95% confidence intervals (95% CI). However, Bonferroni correction was employed to adjust the significance level in the case of multiple comparisons.

## Electronic supplementary material

Supplemental Data
