# Supplementary material for: Variants of the ABCA3 gene might contribute to susceptibility to interstitial lung diseases in the Chinese population
Source: Sci Rep. 2017 Jun 22;7:4097. doi: 10.1038/s41598-017-04486-y (PMC5481373; doi:10.1038/s41598-017-04486-y)
Supplement: Supplementary file 1 — Supplemental Data [file 41598_2017_4486_MOESM1_ESM.pdf]

Supplemental Data

Variants of the ABCA3 gene might contribute to susceptibility to interstitial lung diseases in the Chinese population

Wei Zhou, Yi Zhuang, Jiapeng Sun, Xiaofen Wang, Qingya Zhao, Lizhi Xu and Yaping Wang

Table S1. The seventeen variations detected in the introns sequences adjacent to exons of the *ABCA3* gene

Intron	Nucleotide change	No. of minor allele (n*=60)		Reported MAF†
		Patient	Healthy control	
5	c.319+50 G>A	7	0	rs46725; C=0.1633
6	c.447+119A>G	30	27	rs323059;T=0.0835
6	c.448-252G>A	2	0	rs2240522;T=0.0078
6	c.448-97G>T	30	21	rs323060;C=0.1575
7	c.613+12G>A	5	0	rs777275480;T=0.00002
9	c.991-105C>A	3	0	rs323066; T=0.0917
9	c.991-20C>T	27	24	rs13332547; A=0.1969
12	c.1467+71G>C	1	1	rs79945458; G=0.0148
12	c.1467+130C>T	1	0	rs138143573; A=0.0010
13	c.1612-227C>A	28	31	rs4787273 ; T=0.2997
14	c.1742-116T>C	22	13	rs2240523; G=0.4748
21	c.3004+34C>T	10	2	rs313909; A=0.4742
23	c.3483+26G>A	1	0	rs183047748 ; T=0.0002
25	c.3862+23C>A	1	0	rs544213510 ;A=0.000009
25	c.3862+117G>T	35	15	rs45452892; A=0.3005
28	c.4360-130G>A	27	17	rs2302035; T=0.3405
31	c.4909+13C>A	1	0	rs555551542; T=0.0004

*n: total number of alleles in investigation; †MAF: minor allele frequency (dbSNP http://www.ncbi.nlm.nih.gov/snp/)

Table S2. Primers Used for the Amplification of the *ABCA3* Gene

Primer	Sequence (5' to 3')	Exon
ABCA3-F1	CATGGCTTTTCCCCAAATCC	4+5
ABCA3-R1	AACTCCTCACCCAGCTGCTT	
ABCA3-F2	GCAGGGTGGGTGGTGGGTTT	6
ABCA3-R2	CCTTGGCGGGGACTGTGATG	
ABCA3-F3	TAAATATGATCTGGCGGAAA	7
ABCA3-R3	ACAACAGCAAAGACTTGGAA	
ABCA3-F4	TCCCGTGGGACCACTGAG	8
ABCA3-R4	CACCAAGCCTTTGGACAT	
ABCA3-F5	AGCGTGATGGCTTCTCTCCC	9+10
ABCA3-R5	AGTCCAACCTTCCCCTGGTC	
ABCA3-F6	TTGTGTCCCGTGTAGATGGC	11
ABCA3-R6	CTCTTGTGGTTGGGTGCTCT	
ABCA3-F7	ATAAAACCGGGAAGGGAAAA	12
ABCA3-R7	AAGGAGGCAGGATTGAGGAG	
ABCA3-F8	TTCTCCACAGCAGTGCCTCGAA	13+14
ABCA3-R8	TGGCGCTGAGATGGTGTTAAA	
ABCA3-F9	TGTGTGTCGTGGGTTTCTCC	15
ABCA3-R9	GGCTCCCTTCCTCCAGTTTA	
ABCA3-F10	CAGCTACGTCAAGGAGAGGTT	16+17
ABCA3-R10	CCTGCCTCTTCCCTCTCACAA	
ABCA3-F11	CCTGGTGCTTGTATAGCTGG	18
ABCA3-R11	AGGGTCTAAGAGTGCCGACT	
ABCA3-F12	CGAGGGGGGGTGATGCTTTA	19
ABCA3-R12	GCCCAGTCCTAGGTGGACGG	
ABCA3-F13	AACCATAGTCCCTCCCTCCA	20
ABCA3-R13	CCATTCAAACGCTTCTCCCT	
ABCA3-F14	GCGTCACACAGAACAGCACC	21
ABCA3-R14	GCAGTCAGGAAGGCGAACTC	
ABCA3-F15	TGCTCAGGTGGCAGGGGGTG	22
ABCA3-R15	AGGAGGATGTGGCAGGGGTT	
ABCA3-F16	TGCTCCGTCCCTGACCTTCT	23
ABCA3-R16	AGTTTCCCCTCGTCCCCTTG	
ABCA3-F17	GGGTCTGAGGACCTCCAAAT	24
ABCA3-R17	GGGGCTGGTGAGCATGAACT	
ABCA3-F18	CTGGAGGTGGGTGTGGTGTG	25
ABCA3-R18	CTGAGGCAGGTGGGAGAGTG	
ABCA3-F19	CTGGAGGTGGGTGTGGTGTG	26
ABCA3-R19	TGTGAGTGGCGAGGGCTGTG	
ABCA3-F20	CCACCAGACCTCCCACATCC	27+28
ABCA3-R20	CACCCCTTCAGAGCCTCCCT	

Primer	Sequence (5’ to 3’)	Exon
ABCA3-F21	GCTTCCCTCCAACCCGCCTC	29
ABCA3-R21	GGTCACACCACCACATCCCA	
ABCA3-F22	CTCAGCCTTATTCCCCCACC	30
ABCA3-R22	GAAACTTCCAGTAACCCACA	
ABCA3-F23	AGCATGGGTATCAGGAACAG	31+32
ABCA3-R23	TAGGGGAGAAATGGAAAGTG	
ABCA3-F24	GGCAGGGGTAGGATTGTGGG	33-1
ABCA3-R24	CAGCTCTGGGAAAGTGAACT	
ABCA3-F25	ATGTCTGCATACTCTGGAGT	33-2
ABCA3-R25	GTGTGATTGAAATGTGAAAG	
